# Polypyrimidine Tract-Binding Protein Regulates Enterovirus 71 Translation Through Interaction with the Internal Ribosomal Entry Site

**DOI:** 10.1007/s12250-019-00089-1

**Published:** 2019-02-22

**Authors:** Juemin Xi, Fei Ye, Guanzhou Wang, Wei Han, Zhizhong Wei, Bin Yin, Jiangang Yuan, Boqin Qiang, Xiaozhong Peng

**Affiliations:** 1 Institute of Medical Biology, Chinese Academy of Medical Sciences, and Peking Union Medical College, Kunming, 650118 China; 2 The State Key Laboratory of Medical Molecular Biology, Department of Molecular Biology and Biochemistry, Institute of Basic Medical Sciences, Chinese Academy of Medical Sciences and Peking Union Medical College, Beijing, 100005 China

**Keywords:** EV71, IRES, PTB, Translation

## Abstract

Enterovirus 71 (EV71), a major causative agent of hand, foot, and mouth disease, has caused periodic infection outbreaks in children in the Asia–Pacific region. In order to describe the largely unknown life cycle of EV71, the molecular basis of its virus-host interactions must first be determined. The 5′ untranslated region of EV71 contains a cloverleaf-like structure and internal ribosomal entry site (IRES), which play an important role in transcription and translation of viral protein. We found that polypyrimidine tract-binding protein 1 (PTB) bound to the IRES of EV71. RNA recognition motifs 1 and 2 of PTB were responsible for its binding to the EV71 IRES. Moreover, PTB protein was shuttled from nucleus to cytoplasm after EV71 infection. Additionally, IRES activity and viral protein production were inhibited by PTB knockdown. These results suggest that PTB interacts with the EV71 IRES, and positively regulates viral protein translation.

**Electronic supplementary material:**

The online version of this article (10.1007/s12250-019-00089-1) contains supplementary material, which is available to authorized users.

## Introduction

EV71 belongs to family *Picornairidae*, and has a single-stranded positive-sense RNA genome. First identified in California in 1969, the virus is a newly emerging health threatening pathogen in the Asia–Pacific region, including Japan, Singapore, China, and Korea (McMinn *et al*. 2001; Ryu *et al*. 2010; Shimizu *et al*. 1999; Singh *et al*. 2002). It is reported to be the main cause of hand, foot, and mouth disease (HFMD) in young children (Ooi *et al*. 2010). There have been millions of cases in China, with hundreds of deaths every year since 2008 (Qiu 2008). Currently, two inactivated EV71 vaccines were shown safe and efficacious in phase III trials in China and have recently been approved for vaccination in China (Li *et al*. 2014; Zhu *et al*. 2013, 2014). However, there are presently no effective drugs for clinical treatment of EV71 infection. In order to develop effective treatment for EV71 infection, the molecular mechanism underlying its replication must first be understood.

The EV71 genome encodes four structural proteins (VP1, VP2, VP3, and VP4) and seven nonstructural proteins (2A, 2B, 2C, 3A, 3B, 3C, and 3D), and the 5′UTR of EV71’s genomic RNA contains a cloverleaf-like structure and an IRES region (Shingler *et al*. 2013; Thompson and Sarnow 2003b). It has been reported that most IRES-dependent translation of these proteins requires the binding of canonical initiation factors, as well as IRES trans factors (ITAFs) (Holcik and Sonenberg 2005). ITAFs with multiple RNA-binding domains (RBDs) are known to bind to the IRES region (Faye and Holcik 2015), and many ITAFs have been found to interact with EV71 5′UTR, including heterogeneous nuclear ribonucleoprotein A1 (hnRNP A1), far-upstream element-binding protein 2 (FBP2), far-upstream element-binding protein 1 (FBP1), heterogeneous nuclear ribonucleoprotein K (hnRNP K), and poly(C)-binding protein 1 (PCBP1). HnRNP A1, FBP2, and FBP1 have been shown to regulate the EV71 IRES-related translation initiation activity, while hnRNP K and PCBP1 are required for the efficient synthesis of viral RNA (Huang *et al*. 2011; Lin *et al*. 2008, 2009a, b; Luo *et al*. 2014).

PTB has four RBDs, which are also known as RRMs (RNA recognition motif), is predominantly expressed in the nucleus, and participates in the alternative splicing of many pre-mRNAs (Auweter and Allain 2008; Spellman *et al*. 2005). PTB has been reported to function as ITAFs in several viruses, including poliovirus (PV), encephalomyocarditis virus (EMCV), hepatitis A virus (HAV), and hepatitis C virus (HCV) (Gosert *et al*. 2000; Hellen *et al*. 1994; Hellen and Wimmer 1995; Schmid and Wimmer 1994; Venkatramana *et al*. 2003). PTB can bind to the 5′UTR of the EV71 genome and compete with the negative regulator protein FBP2 for IRES binding (Lin *et al*. 2009a). Preventing the interaction of PTB and the EV71 IRES region through the use of quinacrine inhibits EV71 replication (Wang *et al*. 2013). However, the details of PTB’s interaction with the EV71 IRES and how it affects the EV71 life cycle remain largely unknown.

In this study, biotinylated EV71 IRES RNA probes were used to pull-down the associated cellular proteins in T98G cells. The domains in EV71 IRES which were responsible for PTB binding to the EV71 IRES were studied. And the impact of PTB on EV71 translation initiation and viral production was also assayed. This study aimed to identify the cellular protein interaction with EV71 IRES and reveal the molecular mechanism of this interaction to improve our understanding of the EV71 life cycle, and to identify potential therapeutic targets of EV71 virus infection.

## Materials and Methods

### Cell Lines and Virus

Human glioblastoma cell line T98G, human bone marrow neuroblastoma cell line SH-Sy5Y, and human embryonic kidney cell line 293ET were purchased from the ATCC and cultured according to the recommended guidelines (http://www.atcc.org). Human embryonal rhabdomyosarcoma cells (RD) from the Cell Center of Peking Union Medical College were cultured in Dulbecco’s modified Eagle’s medium (DMEM) with 10% fetal bovine serum (FBS), 100 U/mL penicillin, and 100 μg/mL streptomycin sulfate (Invitrogen, USA). Human astrocyte cell line HA was purchased from ScienCell Research Laboratories and cultured in astrocyte medium (catalog 1801). All cells were maintained at 37 °C in a 5% CO_2_ incubator.

EV71 virus strain (Human enterovirus 71 strain 87-2008 Xi’an Shaanxi, GenBank ID: HM003207.1) was kindly supplied by Professor Wenbo Xu in Chinese Center Disease Control and Prevention. Cells were infected by EV71 and incubated at 37 °C for 1 h. Unbound viruses were washed away by PBS. Virus titration was determined by a modified plaque assay using RD cells (Deng *et al*. 2012). Generally, RD cells (2 × 10^5^ cells) were seeded onto a 12-well plate, incubated overnight, and then infected with serially diluted virus suspension. After incubation for 1 h, the virus suspension was replaced with DMEM containing 2% FBS and 0.8% methylcellulose. After 96 h post-infection, the medium was removed. The cells were fixed with 10% formaldehyde and stained with 1% crystal violet. The virus titer was expressed as PFU (plaque forming unit) per mL.

### Plasmids, Small Interfering RNAs, and Antibodies

To make full-length and truncated PTB expression constructs, cDNA from T98G cells was used as template for amplification by specific primers (Supplementary Table S1). The resultant PCR products were cloned into pcDNA4.0 vector (Invitrogen, USA). The secondary structure of EV71 5′UTR was predicted by the mfold web server and used to design full-length and truncated IRES probes (Bellaousov *et al*. 2013), which were then amplified from EV71 cDNA using specific primers (Supplementary Table S1). The EV71 IRES element contains five major stem-loops (SL II–VI). The PCR products were cloned into pGEM-3zf vector (Promega, USA) to generate pGEM-3zf-IRES, pGEM-3zf-SL II (121–180), SL III (190–230), SL IV (241–450), SL V (451–563), SLVI (564–742, stem-loop VI, and linker region), respectively. All the constructs were confirmed by DNA sequencing.

The siRNAs against PTB and negative control siRNA (siNC) were purchased from Invitrogen (Cat. AM16708). Antibodies used in this study were as follows: rabbit anti-PTB polyclonal (generated in our lab) antibody, mouse anti-β-actin monoclonal antibody (1:3000, Sigma, USA), mouse anti-CREB monoclonal antibody (1:1000, CST, USA), mouse anti-his monoclonal antibody (1:1000, Qiagen, Germany), rabbit anti-EV71 VP1 polyclonal antibody (1:1000, Abnova, Taiwan, China), and mouse anti-EV713C monoclonal antibody (1:200, Millipore, USA).

### *In vitro* Transcription

The plasmids of pGEM-3zf-IRES and six IRES truncation variants were linearized with Hind III restriction enzyme. EV71 IRES RNA was synthesized using a T7 transcription kit and with biotin NTP (Takara, Japan, GE Healthcare, USA) following the manufacture’s protocol. The RNAs were purified using mini quick spin RNA columns (Roche, USA) and analyzed on 1% agarose gels.

### Biotinylated RNA Pull-Down Assays

T98G, HA, RD, and SH-SY5Y cells were pelleted, and then prepared using nuclear protein extraction reagents (Thermo, USA). A Bio-Rad protein assay was used to determine the protein concentrations of cell extracts. The RNA pull-down assay protocol was modified from method previously reported (Lin *et al*. 2008). Biotinylated EV71 IRES was used to capture interacting proteins. Bands of interest were excised, digested in gel with trypsin, and identified by Bruker Ultraflex matrix-assisted laser desorption/ionization time-of-flight mass spectrometry (MALDI-TOF MS).

### Database-Searching Algorithm

Mass data were exported to the Biotool 2.0 software package, and the Mascot (http://www.matrixscience.com) algorithm was used to identify the proteins.

### Co-immunoprecipitation and RT-PCR

T98G cell extract was prepared and used for co-immunoprecipitation after 6 h of EV71 infection at a multiplicity of infection (MOI) of 20. Lysates were pre-cleaned by incubation with protein A-agarose beads (50% in lysis buffer) on ice for 1 h. After centrifugation at 10,000 ×*g* at 4 °C for 10 min, the supernatants were used for the immunoprecipitation assay. 100 μL of lysate, 400 μL of lysis buffer, and 10 μL of PTB antibody were mixed together and incubated on ice for 2 h. Pre-washed protein A-agarose beads (v/v, 50% in PBS) were added into the mixtures and incubated on ice for another 1 h. Mixtures were pelleted by centrifugation at 1000 ×*g* at 4 °C for 5 min and washed five times with lysis buffer. Each reaction mixture was resuspended in 400 μL of proteinase K buffer (100 mmol/L Tris–HCl, pH 7.5, 12.5 mmol/L EDTA, 150 mmol/L NaCl, 1% SDS, and 100 μg of proteinase K) and incubated for 30 min at 37 °C. RNA was extracted with phenol/chloroform. RT-PCR was performed, with the fragments amplified using primers specific to either the EV71 5′UTR or to the ribosomal protein S16 (Supplementary Table S1).

### Immunofluorescence Microscopy

T98G cells were seeded on 20-mm cover slips to 90% confluence in 24 wells and infected with EV71 at MOI of 5. Culture medium was removed, and the cells were washed three times with PBS 6 h post-infection, then fixed with 4% PFA for 20 min at room temperature. Cells were blocked in PBS containing 5% BSA for 1 h at 37 °C and incubated with antibodies at 4 °C overnight. Next, the cells were washed three times with PBS, and incubated with secondary antibody: either donkey anti-mouse antibody labeled with Alexa Fluor 488 (CA-21202, Invitrogen), or donkey anti-rabbit antibody labeled with Alexa Fluor 594 (A-21207, Invitrogen). Images were captured using an Olympus microscope and digital camera system (BX51 and DP70, or BX53 and DP72, Olympus).

### Dicistronic Expression Assay

T98G cells were transfected with either PTB siRNA or pcDNA4.0-PTB. After two days, dicistronic construct pRF or pRF-EV71 IRES was co-transfected with siRNA duplexes into T98G cells. Forty-eight hours later, *Renilla* luciferase (Rluc) and firefly luciferase (Fluc) activities were determined in a LB9507 bio-luminometer using a dual-luciferase reporter assay (Promega, USA) according to the manufacturer’s instructions. PTB expression was determined by Western blotting of cell lysates.

### Western Blot Assays

Cells were harvested and washed three times with PBS, and then incubated with TNTE lysis buffer (50 mmol/L Tris–HCl, pH 7.4, 150 mmol/L NaCl, 1 mmol/L EDTA, 10 mmol/L sodium pyrophosphate, 0.5% Triton X-100, 1 mmol/L sodium vanadate, and 25 mmol/L sodium fluoride) supplemented with protease inhibitors (2 mg/mL aprotinin, 2 mg/mL pepstain, 2 mg/mL leupeptin and 2 mg/mL PMSF) on ice for 30 min to extract cells proteins. Lysates were centrifugated at 10,000 ×*g* at 4 °C for 30 min, after which the supernatants were boiled with SDS-PAGE sample buffer for 10 min. The cell lysates were electrophoresed on a 12% SDS-PAGE, and transferred to nitrocellulose membranes (Amersham, USA). The membranes were blocked with Tris-buffered saline containing 0.2% (v/v) Tween-20 and 5% non-fat dry milk, and incubated with the indicated antibodies. Blots were visualized by an x-ray film (Kodak, USA).

### Statistical Analysis

All experiments were reproducible, and each assay was repeated at least three times. The data were analyzed using a two-tailed paired Student’s *t* test and values with *P* < 0.05 were considered statistically significant.

## Results

### PTB Interacts with EV71 Viral RNA in IRES Region

**Fig. 1:**
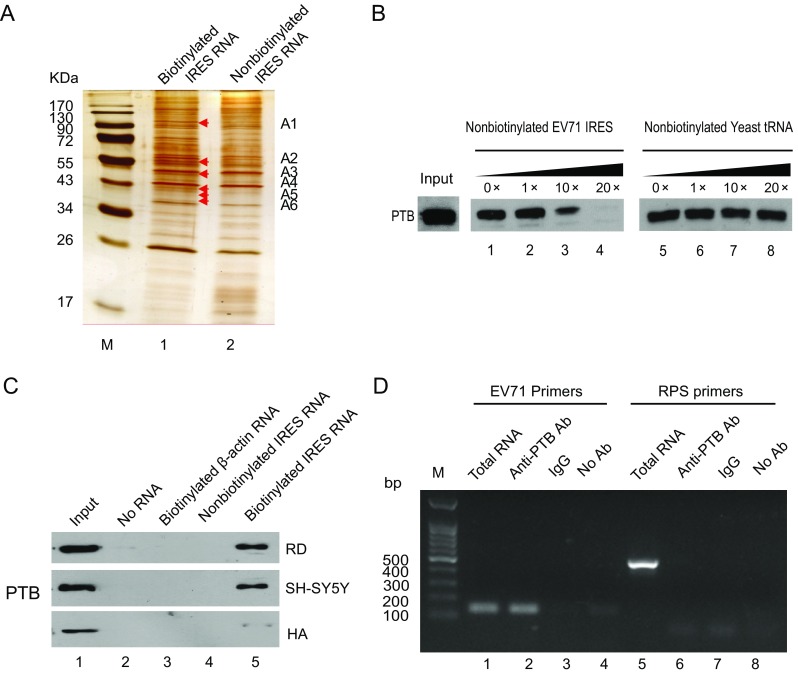
Interaction of PTB with the EV71 IRES region. **A** Pull down of proteins with the EV71 IRES in T98G cell lysate. The specific bands interacting with EV71 IRES are shown by silver staining. Lane 1: biotinylated IRES RNA, Lane 2: nonbiotinylated EV71 IRES RNA. **B** A specific association between PTB and the EV71 IRES region was confirmed by Western blotting and a competition assay. Increasing amounts of unlabelled RNA were added to compete with the biotin-labeled EV71 IRES RNA interacting with PTB. The eluted proteins were separated by 12% SDS-PAGE. Lanes are as follows: lanes 1–4, unlabeled EV71 IRES RNA; lanes 5–8, unlabeled yeast tRNA. **C** Extracts of RD cells, SH-SY5Y cells, and HA cells were prepared and then incubated without RNA (lane 2), with biotinylated actin RNA (lane 3), non-biotinylated EV71 IRES RNA (lane 4), or biotinylated EV71 IRES RNA (lane 5). After pull-down assay, the bound proteins were eluted, boiled, and subjected to 12% SDS-PAGE. PTB protein was detected by western blot with a rabbit anti-PTB antibody. The inputs were cell extracts of RD, SH-SY5Y and HA (lane 1). **D** EV71 IRES RNA was pulled down with PTB from EV71-infected T98G cell lysate. T98G cells were infected with EV71 at an MOI of 20 for 6 h and then cell extracts were incubated with rabbit anti-PTB antibody (lanes 2 and 6), normal rabbit IgG (lanes 3 and 7), or without antibody (− Ab) (lanes 4 and 8). Following washing and dissociation, the RNA extract was prepared and subjected to RT-PCR analysis with primers specific for the ribosomal protein S16 (PRS16) RNA (lanes1–4) or for EV71 IRES region RNA (lanes 5–8). Lane 1, cell lysate without immunoprecipitation as a positive RT-PCR control; Lane 2, anti-PTB antibody incubated with 200 mg infected-cell lysate; Lane 3, negative control with rabbit IgG; Lane 4, negative control with no antibody.

To identify proteins that interact with the IRES of EV71, biotinylated EV71 IRES probes were used to capture the interacting proteins. As shown in Fig. 1A, compared with nonbiotinylated RNA (lane 2), the biotinylated EV71 IRES RNA enriched 6 differential bands (lane 1). Using MALDI-TOF MS analysis, 12 proteins were identified, of which PTB, poly(rC)-binding protein 2 (PCBP2), and proliferation-associated protein 2G4 (ITAF45) have been found to interact with the IRES of picornavirus (Table 1). PTB has been found to be an important protein during the life cycle of enterovirus. However, the details of PTB’s interaction with the EV71 IRES remain to be determined.

**Table 1:** MALDI-TOF MS results of cellular proteins associated with the EV71 IRES.

Band no	NCBI accession no	Protein name
A1	gi|4826998	Splicing factor, proline- and glutamine-rich [*Homo sapiens*]
	gi|1616766	Cationic trypsinogen [*Homo sapiens*]
A2	gi|4929579	CGI-55 protein [*Homo sapiens*]
	gi|4506243	Polypyrimidine tract-binding protein 1 isoform a [*Homo sapiens*]
A3	gi|124494254	Proliferation-associated protein 2G4 [*Homo sapiens*]
	gi|4503481	Elongation factor 1-gamma [*Homo sapiens*]
	gi|693933	2-phosphopyruvate-hydratase alpha-enolase [*Homo sapiens*]
	gi|1199487	Collagen binding protein 2 [*Homo sapiens*]
A4	gi|28336	Mutant beta-actin (beta’-actin) [*Homo sapiens*]
	gi|181914	DNA-binding protein [*Homo sapiens*]
	gi|14141166	Poly(rC)-binding protein 2 isoform b [*Homo sapiens*]
A5	gi|181914	DNA-binding protein [*Homo sapiens*]
A6	gi|14141166	Poly(rC)-binding protein 2 isoform b [*Homo sapiens*]

To elucidate the specific interaction of PTB and the EV71 IRES region, a competition assay and Western blotting were carried out. The specific interaction was outcompeted by non-biotinylated EV71 IRES (Fig. 1B, lanes 1–4), but not by non-biotinylated yeast tRNA (Fig. 1B, lanes 5–8). To confirm this interaction, additional cell lines were assayed. RNA pull-down and Western blot results showed that PTB interacted with EV71 IRES in all of the cell lines tested, including the RD, SH-SY5Y, and HA cell lines (Fig. 1C). Non-biotinylated IRES RNA or biotinylated β-actin probe were used as negative control.

To further confirm the results in infected cells, PTB antibody was used to pull-down the EV71 IRES. EV71 IRES was detected in total RNA extract and in the assay with PTB antibody, but not with rabbit IgG or in the absence of antibody (Fig. 1D). Primers specific to ribosomal protein S16 (RPF16) were used as negative control. These results illustrated that PTB interacts with EV71 IRES region.

### PTB Binds to the Stem-loop VI within the EV71 IRES Region

**Fig. 2:**
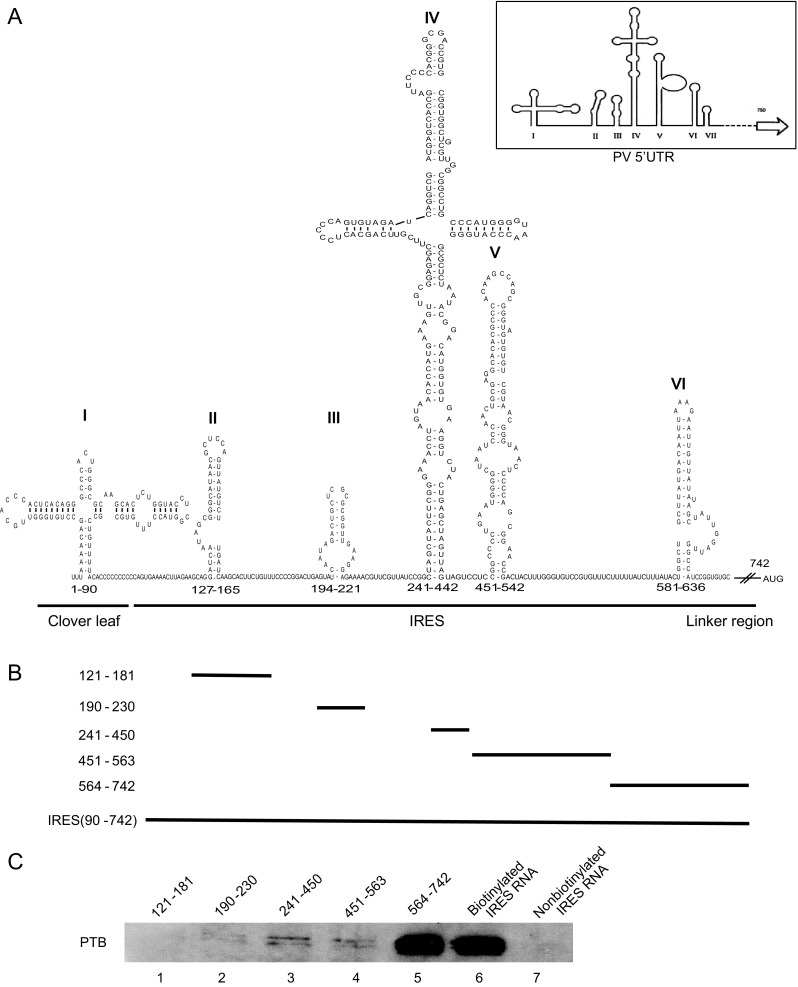
Determination of the EV71 IRES sequences required for the binding of PTB. **A** Prediction of the RNA secondary structure of the EV71 5′UTR by the mfold web serve. The modified schematic representation of the secondary structure of poliovirus (PV) 5′UTR (modified from Hellen *et al*. 1994) is shown in the top right corner. **B** Plasmids carrying different deletions in the five stem-loops of EV71 IRES: pGEM-3zf-(SL II, 121–181), (SL III, 190–230), (SL IV, 241–450), (SL V, 451–463), (SL VI, 564–742). **C** Analysis of the regions responsible for the interaction in the EV71 IRES region using various truncated RNA forms, transcribed *in vitro* and biotinylated. T98G cell lysate were incubated with these biotin-labeled RNAs and the non-biotinylated IRES RNA probes were used as controls. After being pulled down by streptavidin, the protein complex was separated by SDS-PAGE and Western blot was carried out to detect PTB in the pulled-down complex (lanes 1–7).

To identify the binding region of EV71 IRES with PTB, the secondary structure of EV71 5′UTR was predicted, and found to be conserved in picornavirus (Fig. 2A). Three functional regions including cloverleaf-like (CL), IRES, and linker regions, were identified based on a previous report (Lin *et al*. 2008). Stem-loops II–VI, which are important for initiation of virus translation, were contained in the EV71 5′UTR. To map the regions of interaction between PTB and EV71 IRES, six different stem-loop RNA probes were generated, including SL II (nt 121–180), SL III (nt 190–230), SL IV (nt 241–450), SL V (nt 451–563), SL VI (nt 564–742), and the entire IRES (nt 90–742) (Fig. 2B). After RNA pull-down assay, Western blot results showed that PTB specially binds to the SL VI (stem-loop VI and linker region) and whole IRES, but not other SLs (Fig. 2C).

### RRM1–2 of PTB Interacts with EV71 IRES

**Fig. 3:**
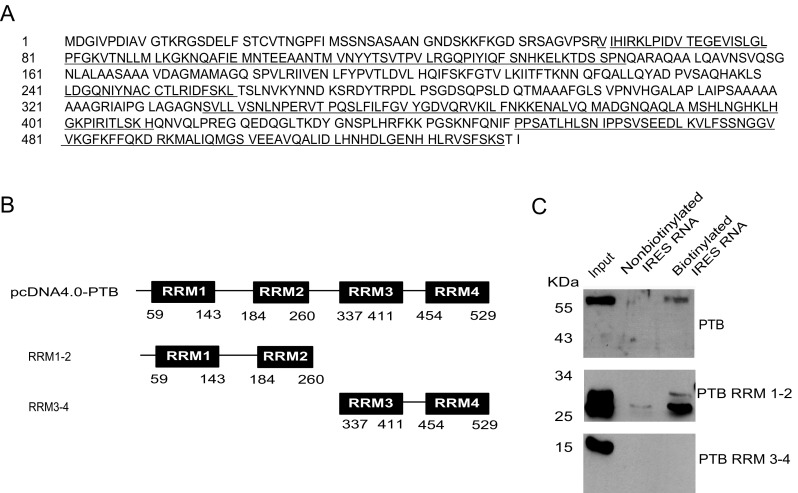
Analysis of the expression of PTB in neuro-related cells and RRMs required for its binding to EV71 IRES. **A** The RRM domains of full-length PTB protein as described. The amino acids of the four RRM domains, RRM1, RRM2, RRM3, and RRM4, are underlined. **B** Three constructed plasmids expressing the full-length PTB, or truncated PTB proteins in which RRM3–4 was deleted (RRM1–2), or RRM1–2 was deleted (RRM3–4), respectively. **C** RNA–protein binding assay showing PTB protein as determined by Western blot analysis using anti-His antibody, from 293ET cells transfected with plasmids coding the His-Tagged proteins, pcDNA4.0-PTB, RRM1–2, RRM3–4, respectively.

PTB protein contains four RRMs (RRM1, RRM2, RRM3, and RRM4), which are important for RNA binding (Clery *et al.*^2008^). The amino acids constituting these four RRM domains are underlined in Fig. 3A. To identify the domains that account for interaction with the EV71 IRES region, three plasmids expressing the one full-length and two truncated PTB proteins (RRM1–2, RRM3–4) were constructed (Fig. 3B). These plasmids were individually transfected into 293ET cells, and the resulting cell lysates were used in pull-down assays with biotinylated EV71 IRES. The results showed that all the three PTB proteins were expressed in transfected cells, and that they were detected in the input lanes. PTB RRM1–2 protein, but not RRM3–4 protein, was detected in the presence of biotinylated EV71 IRES (Fig. 3C). These results indicated that RRM1–2 of PTB is responsible for the binding of PTB to the EV71 IRES.

### PTB Transfers to the Cytoplasm during EV71 Infection

**Fig. 4:**
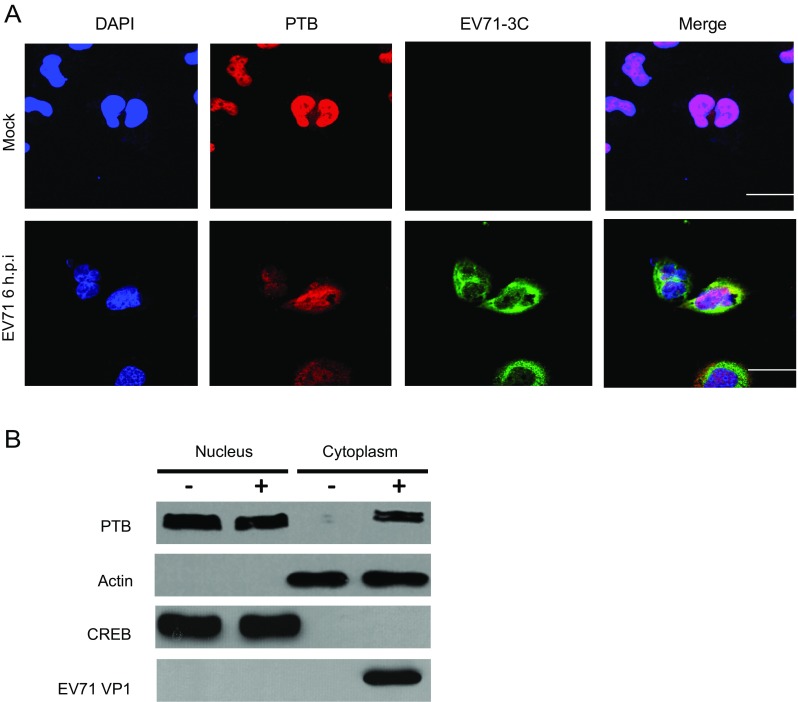
Subcellular distribution of PTB protein during EV71 infection. T98G cells mock-infected (upper panels) or infected with EV71 at an MOI of 20 (lower panels). At 6 h post infection, cells were fixed with formaldehyde, washed, and detected with antibody against PTB or EV71 3C protein. FITC-conjugated goat anti-rabbit IgG or TRITC-conjugated goat anti-mouse IgG was used as secondary antibody and stained with DAPI. Images were captured by confocal laser scanning microscopy. PTB (red), EV71 3C (green), DAPI (blue). Bar = 10 µm. **B** T98G cells infected with or without EV71 at an MOI of 20 for 6 h. Whole cell lysate, cytoplasm, and nucleus extractions were prepared from mock infected and EV71-infected cells, respectively. Endogenous proteins as detected by Western blot analysis using antibodies to CREB, EV71 VP1, PTB, or β-actin. The blot is representative of three independent experiments with similar results.

PTB is localized mainly in the nucleus, but can shuttle between nucleus and cytoplasm to complete different functions (Xie *et al*. 2003). To further illustrate whether EV71 infection induces changes in PTB localization, T98G cells were infected with EV71, and the localization of PTB was monitored. Confocal microscopy demonstrated that PTB was mainly expressed in the nucleus in mock cells; however, after EV71 infection, a portion of the PTB proteins were shuttled from the nucleus to the cytoplasm (Fig. 4A). To further confirm this changed expression pattern, the PTB expression pattern during EV71 infection was examined. The results showed that partial PTB protein was translocated into the cytoplasm in EV71-infected cells, while PTB was expressed exclusively in the nuclei in non-infected cells (Fig. 4B). Consistent with the confocal results, EV71 VP1 protein was exclusively located in the cytoplasm. The positive controls, β-actin and CREB, were exclusively expressed in the cytoplasm and nucleus, respectively. These results illustrated that, upon EV71 infection, PTB translocated from the nucleus to the cytoplasm, implying that PTB might be recruited by EV71 to participate the viral life cycle.

### PTB Promotes IRES-Dependent Translation

**Fig. 5:**
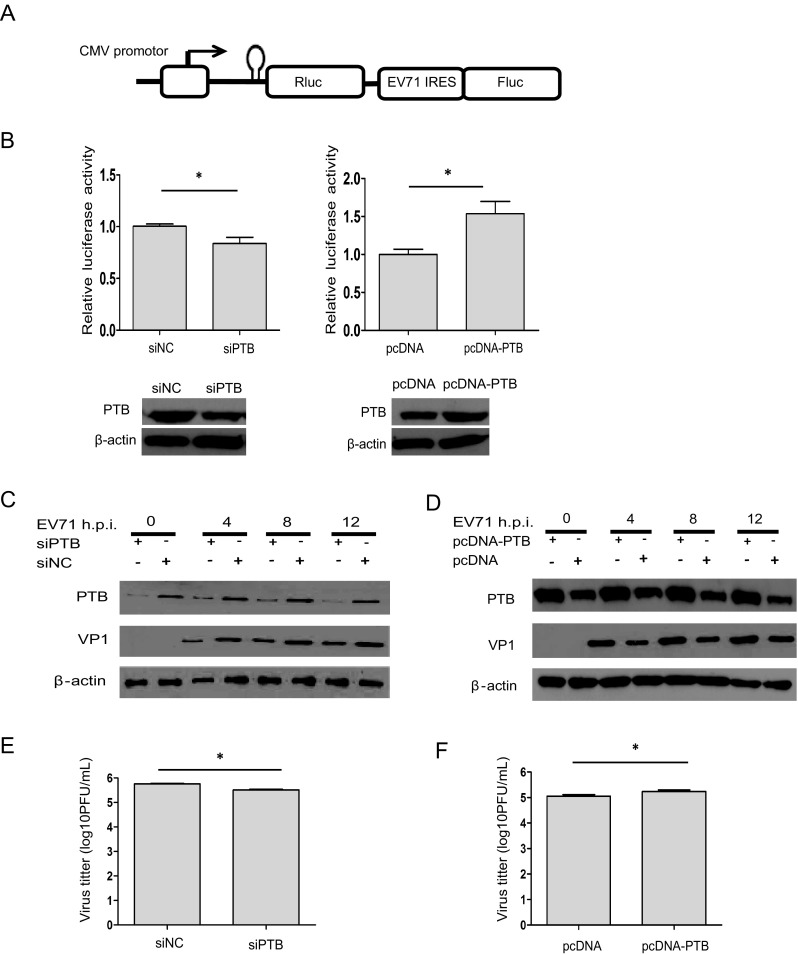
Analysis of PTB in the regulation of EV71 viral translation and virus production. **A** Schematic diagram of dicistronic reporter plasmids pRF-EV71 IRES. **B** T98G cells were transfected with either PTB siRNA or pcDNA4.0-PTB. 48 h later, dicistronic construct pRF or pRF-EV71 IRES and siRNA duplexes were co-transfected into T98G cells. After 48 h, the relative ISRE activity were analyzed by monitoring the luciferase activity. Western blot were used to analyze the expression level of PTB and β-actin. **C**–**F** T98G cells were transfected with siRNA targeting PTB (siPTB) and control siRNA (siNC), respectively. 48 h later, T98G cells were transfected with plasmids containing either full-length PTB cloned into pcDNA4.0 vector (pcDNA-PTB), or vector without PTB (pcDNA). After 24 h, cells were infected with EV71 at an MOI of 20 for 4, 8 and 12 h. Cell culture supernatants from EV71-infected cells were prepared and subjected to plaque assays. Western blotting was used to determine the expression level of EV71 VP1 protein, PTB or β-actin protein. **E**, **F** The effects of PTB on EV71 growth. Cell culture supernatants from **C** and **D** were determined by plaque assay. Each bar represents the average of triplicate data points with the standard deviation represented as the error bar. *P < 0.05 and **P < 0.01 versus negative control.

Cap-independent initiation of translation is a major pathway for picornavirus translation, and IRES provides an alternative means of viral polyprotein translation initiation. To evaluate the effects of PTB on EV71 IRES activity, a dicistronic reporter plasmid was used (Fig. 5A). The first cistronic (*Renilla* luciferase, Rluc) is cap-dependent, while the translation of the second cistronic (Firefly luciferase, Fluc) is dependent on EV71 IRES activity. One stem loop was added upstream of Rluc to prevent a high Rluc background signal arising from false initiation of translation. The Ratio of Fluc to Rluc expression could be considered as the relative IRES activity. Compared with the siNC negative control, knockdown of PTB decreased EV71 IRES activity to 70% (*P* < 0.05) (Fig. 5B), while overexpression of PTB increased EV71 IRES activity to 160% (*P* < 0.05). These results suggest that PTB can positively regulate EV71 IRES activities related to initiation of translation.

### PTB Promotes EV71 Viral Protein Expression and Virus Production

To study the roles of PTB in virus translation, the expression level of EV71 VP1 was examined. The expression level of EV71 VP1 was decreased in PTB knockdown cells at 4 h, 8 h, and 12 h post-EV71 infection, respectively (Fig. 5C), while the expression level of EV71 VP1 was increased in PTB-overexpression cells during infection (Fig. 5D), indicating that PTB promotes the expression of viral protein.

The effects of PTB on EV71 growth were also evaluated. As illustrated in Fig. 5E, 5F, virus production and viral titer in EV71-infected cells were enhanced by overexpression of PTB, but reduced by knockdown of PTB, suggesting that PTB positively regulates viral replication.

## Discussion

Translation initiation of many RNA virus genomes is cap-independent, but IRES-dependent in viral genomic RNA 5′UTR. Viral IRESs contain four major structure groups, which depend on different initiation of translation patterns, with EV71 containing a type I IRES element (Jackson *et al*. 2010; Thompson and Sarnow 2003a). The interactions between viral IRES and ITAFs are crucial for understanding initiation of viral translation. ITAFs play an important role in regulating IRES-related translation initiation (Hung *et al*. 2016; Lin *et al*. 2008; Lu *et al*. 2004; Tolbert *et al*. 2017). The molecular mechanisms underlying interactions between ITAFs and EV71 IRES remain poorly understood. In this study, using a biotinylated RNA–protein pull-down assay combined with MALDI-TOF analysis, a total of 12 different proteins associated with the EV71 IRES were shown to bind to the EV71 IRES (Table 1). PTB, PCBP2, and ITAF45 have been implicated in the initiation on Type I picornavirus IRESs (Sweeney *et al*. 2014). The interaction of PTB with the EV71 IRES region was demonstrated to be common to different cell lines, but relatively weak in HA cells. It has been reported that PTB expression was also strongly increased in malignant glioblastoma multiforme tumors relative to adjacent normal tissue (Wei *et al*. 2000). We speculate that PTB expression may be lower in HA cells compared to other cell lines. Other proteins such as SFPQ, cationic trypsinogen, and CGI-55 protein were recently found to bind to the EV71 IRES region. Further investigation into the roles of the interactions between these identified proteins and EV71 IRES in EV71 life cycle is needed.

PTB belongs to the hnRNP family, and preferentially binds to pyrimidine-rich sequences of RNA (Ghetti *et al*. 1992). PTB has multiple functions, which include alternative splicing, polyadenylation, stabilization, localization, and IRES-mediated translation of cellular and viral genes (Black 2003; Knoch *et al*. 2004; Sawicka *et al*. 2008; Tischendorf *et al*. 2004). PTB has also been reported to participate in translation initiation in many viruses such as poliovirus, EMCV, HRV14 and FMDV (Hellen *et al*. 1993; Niepmann 1996; Rojas-Eisenring *et al*. 1995; Witherell *et al*. 1993). In the present study, the expression of EV71 VP1 was decreased in cells with PTB knockdown, and increased in cells in which PTB was overexpressed during infection, suggesting a regulation cascade through the binding of PTB to the EV71 IRES. The predicted secondary structure of the EV71 5′UTR contained a cloverleaf-like region, IRES, and linker region. *Picornavirus* IRES elements are known to contain conserved secondary structures.

In poliovirus, three noncontiguous PTB-binding sites have been identified using UV cross-linking assays, and reported to be located within the 5′UTR, between nt 70 and 288, and 443 and 539 (domain V), and 630 and 730 (linker region) (Hellen *et al*. 1994). PTB binds to the basal half of domain V and stimulates the initiation of translation by modulating eIF4G binding (Kafasla *et al*. 2010). In our study, a panel of RNA transcripts corresponding to the stem-loop segments of EV71 IRES was generated. Using an RNA pull-down assay, we found that PTB acted as an ITAF and interacted with the 564–742 nt of the EV71 IRES region, which might form stem-loop VI and a linker region within the 5′UTR, and was important for the initiation of viral translation. Stem-loop VI of EV71 was reported to be the main region bound by ribosomal preinitiation complexes (Sweeney *et al*. 2014). Whether the linker region within EV71 5′UTR alone can interact with PTB, as in poliovirus, needs to be studied further.

In addition to loop VI of IRES region, PTB also interacts with 1–90 nt of viral RNA 5′UTR, implying that PTB may take part in viral RNA replication. Quinacrine negatively regulates EV71 infection by inhibiting the interactions between PTB and the EV71 5′UTR region (Wang *et al*. 2013), indicating a potential target against EV71 infection. Furthermore, the regions on which quinacrine acts to impair EV71 replication should be studied further. 3′UTR and the cloverleaf-like structure were reported to be crucial for virus replication, but whether PTB affects EV71 transcription by binding to these parts requires further investigation.

PTB has four RRMs, with flexible linkers between RRM 1 and 2, and between RRM 2 and 3. RRM 3 and 4 appear to act as a di-domain separated by a short linker (Oberstrass *et al*. 2005). In poliovirus, IRES activation was abrogated by the mutation of RRM1, 2, and 4, while RRM 3 appears to have few functions (Kafasla *et al*. 2011). Here, we found that the interaction domain of PTB with the EV71 5′UTR was mainly located in the RRM1–2 region (Fig. 3), which was revealed to be important for the interaction between PTB and the EV71 IRES, either directly or together with other cellular proteins. However, identification of the domain that plays the critical role in stimulating EV71 IRES initiation requires further research.

PTB protein is mostly accumulated in the nuclear compartment, while the EV71 life cycle mainly plays out in the cell cytoplasm. The mechanism by which PTB and the virus interact is worthy of further exploration. Viruses rely on their hosts for replication and for the maintenance of viability, and the two become engaged in a dynamic interaction during infection. Previous research has revealed that viruses can reorganize the host’s secretory pathway and induce the formation of specialized organelles for viral RNA replication (Hsu *et al*. 2010). In DENV infection, PTB translocated noticeably from the nucleus to the cytoplasm (Agis-Juarez *et al*. 2009). In our immunofluorescence and Western blotting experiments, a significant portion of total PTB location was found in cytoplasm following EV71 infection. To our knowledge, it is the first evidence that PTB proteins translocate to the cytoplasm during EV71 infection, and suggests an important role for PTB in the EV71 viral life cycle. With knockdown of PTB, both the expression of EV71 VP1 and the viral titer were decreased. In contrast, when PTB was overexpressed, EV71 VP1 expression and the viral titer were both increased (Fig. 5), indicating that PTB positively regulated viral production.

In conclusion, our results reveal that PTB functions as an ITAF and promotes EV71 IRES-related translation as well as virus production. Our findings allow for improved understanding of the function of PTB in EV71 IRES-related translation and suggest potential antiviral targets in EV71 infection.

## Electronic Supplementary Material

Below is the link to the electronic supplementary material.

Supplementary material 1 (PDF 80 kb)

## References

[CR1] Agis-Juarez RA, Galvan I, Medina F, Daikoku T, Padmanabhan R, Ludert JE, del Angel RM (2009). Polypyrimidine tract-binding protein is relocated to the cytoplasm and is required during dengue virus infection in Vero cells. J Gen Virol.

[CR2] Auweter SD, Allain FH (2008). Structure-function relationships of the polypyrimidine tract binding protein. Cell Mol Life Sci.

[CR3] Bellaousov S, Reuter JS, Seetin MG, Mathews DH (2013). RNAstructure: Web servers for RNA secondary structure prediction and analysis. Nucleic Acids Res.

[CR4] Black DL (2003). Mechanisms of alternative pre-messenger RNA splicing. Annu Rev Biochem.

[CR5] Clery A, Blatter M, Allain FH (2008). RNA recognition motifs: boring? Not quite. Curr Opin Struct Biol.

[CR6] Deng JX, Nie XJ, Lei YF, Ma CF, Xu DL, Li B, Xu ZK, Zhang GC (2012). The highly conserved 5′ untranslated region as an effective target towards the inhibition of Enterovirus 71 replication by unmodified and appropriate 2′-modified siRNAs. J Biomed Sci.

[CR7] Faye MD, Holcik M (2015). The role of IRES trans-acting factors in carcinogenesis. Biochim Biophys Acta.

[CR8] Ghetti A, Pinol-Roma S, Michael WM, Morandi C, Dreyfuss G (1992). hnRNP 1, the polyprimidine tract-binding protein: distinct nuclear localization and association with hnRNAs. Nucleic Acids Res.

[CR9] Gosert R, Chang KH, Rijnbrand R, Yi M, Sangar DV, Lemon SM (2000). Transient expression of cellular polypyrimidine-tract binding protein stimulates cap-independent translation directed by both picornaviral and flaviviral internal ribosome entry sites in vivo. Mol Cell Biol.

[CR10] Hellen CU, Wimmer E (1995). Translation of encephalomyocarditis virus RNA by internal ribosomal entry. Curr Top Microbiol Immunol.

[CR11] Hellen CU, Witherell GW, Schmid M, Shin SH, Pestova TV, Gil A, Wimmer E (1993). A cytoplasmic 57-kDa protein that is required for translation of picornavirus RNA by internal ribosomal entry is identical to the nuclear pyrimidine tract-binding protein. Proc Natl Acad Sci U S A.

[CR12] Hellen CU, Pestova TV, Litterst M, Wimmer E (1994). The cellular polypeptide p57 (pyrimidine tract-binding protein) binds to multiple sites in the poliovirus 5′ nontranslated region. J Virol.

[CR13] Holcik M, Sonenberg N (2005). Translational control in stress and apoptosis. Nat Rev Mol Cell Biol.

[CR14] Hsu NY, Ilnytska O, Belov G, Santiana M, Chen YH, Takvorian PM, Pau C, van der Schaar H, Kaushik-Basu N, Balla T, Cameron CE, Ehrenfeld E, van Kuppeveld FJ, Altan-Bonnet N (2010). Viral reorganization of the secretory pathway generates distinct organelles for RNA replication. Cell.

[CR15] Huang PN, Lin JY, Locker N, Kung YA, Hung CT, Huang HI, Li ML, Shih SR (2011). Far upstream element binding protein 1 binds the internal ribosomal entry site of enterovirus 71 and enhances viral translation and viral growth. Nucleic Acids Res.

[CR16] Hung CT, Kung YA, Li ML, Brewer G, Lee KM, Liu ST, Shih SR (2016). Additive promotion of viral internal ribosome entry site-mediated translation by far upstream element-binding protein 1 and an enterovirus 71-induced cleavage product. PLoS Pathog.

[CR17] Jackson RJ, Hellen CU, Pestova TV (2010). The mechanism of eukaryotic translation initiation and principles of its regulation. Nat Rev Mol Cell Biol.

[CR18] Kafasla P, Morgner N, Robinson CV, Jackson RJ (2010). Polypyrimidine tract-binding protein stimulates the poliovirus IRES by modulating eIF4G binding. EMBO J.

[CR19] Kafasla P, Lin H, Curry S, Jackson RJ (2011). Activation of picornaviral IRESs by PTB shows differential dependence on each PTB RNA-binding domain. RNA.

[CR20] Knoch KP, Bergert H, Borgonovo B, Saeger HD, Altkruger A, Verkade P, Solimena M (2004). Polypyrimidine tract-binding protein promotes insulin secretory granule biogenesis. Nat Cell Biol.

[CR21] Li R, Liu L, Mo Z, Wang X, Xia J, Liang Z, Zhang Y, Li Y, Mao Q, Wang J, Jiang L, Dong C, Che Y, Huang T, Jiang Z, Xie Z, Wang L, Liao Y, Liang Y, Nong Y, Liu J, Zhao H, Na R, Guo L, Pu J, Yang E, Sun L, Cui P, Shi H, Wang J, Li Q (2014). An inactivated enterovirus 71 vaccine in healthy children. New Engl J Med.

[CR22] Lin JY, Li ML, Huang PN, Chien KY, Horng JT, Shih SR (2008). Heterogeneous nuclear ribonuclear protein K interacts with the enterovirus 71 5′ untranslated region and participates in virus replication. J Gen Virol.

[CR23] Lin JY, Li ML, Shih SR (2009). Far upstream element binding protein 2 interacts with enterovirus 71 internal ribosomal entry site and negatively regulates viral translation. Nucleic Acids Res.

[CR24] Lin JY, Shih SR, Pan M, Li C, Lue CF, Stollar V, Li ML (2009). hnRNP A1 interacts with the 5′ untranslated regions of enterovirus 71 and Sindbis virus RNA and is required for viral replication. J Virol.

[CR25] Lu H, Li W, Noble WS, Payan D, Anderson DC (2004). Riboproteomics of the hepatitis C virus internal ribosomal entry site. J Proteome Res.

[CR26] Luo Z, Dong X, Li Y, Zhang Q, Kim C, Song Y, Kang L, Liu Y, Wu K, Wu J (2014). PolyC-Binding protein 1 interacts with 5′-untranslated region of enterovirus 71 RNA in membrane-associated complex to facilitate viral replication. PLoS ONE.

[CR27] McMinn P, Stratov I, Nagarajan L, Davis S (2001). Neurological manifestations of enterovirus 71 infection in children during an outbreak of hand, foot, and mouth disease in Western Australia. Clin Infect Dis.

[CR28] Niepmann M (1996). Porcine polypyrimidine tract-binding protein stimulates translation initiation at the internal ribosome entry site of foot-and-mouth-disease virus. FEBS Lett.

[CR29] Oberstrass FC, Auweter SD, Erat M, Hargous Y, Henning A, Wenter P, Reymond L, Amir-Ahmady B, Pitsch S, Black DL, Allain FH (2005). Structure of PTB bound to RNA: specific binding and implications for splicing regulation. Science.

[CR30] Ooi MH, Wong SC, Lewthwaite P, Cardosa MJ, Solomon T (2010). Clinical features, diagnosis, and management of enterovirus 71. Lancet Neurol.

[CR31] Qiu J (2008). Enterovirus 71 infection: a new threat to global public health?. Lancet Neurol.

[CR32] Rojas-Eisenring IA, Cajero-Juarez M, del Angel RM (1995). Cell proteins bind to a linear polypyrimidine-rich sequence within the 5′-untranslated region of rhinovirus 14 RNA. J Virol.

[CR33] Ryu WS, Kang B, Hong J, Hwang S, Kim A, Kim J, Cheon DS (2010). Enterovirus 71 infection with central nervous system involvement, South Korea. Emerg Infect Dis.

[CR34] Sawicka K, Bushell M, Spriggs KA, Willis AE (2008). Polypyrimidine-tract-binding protein: a multifunctional RNA-binding protein. Biochem Soc Trans.

[CR35] Schmid M, Wimmer E (1994). IRES-controlled protein synthesis and genome replication of poliovirus. Arch Virol Suppl.

[CR36] Shimizu H, Utama A, Yoshii K, Yoshida H, Yoneyama T, Sinniah M, Yusof MA, Okuno Y, Okabe N, Shih SR, Chen HY, Wang GR, Kao CL, Chang KS, Miyamura T, Hagiwara A (1999). Enterovirus 71 from fatal and nonfatal cases of hand, foot and mouth disease epidemics in Malaysia, Japan and Taiwan in 1997–1998. Jpn J Infect Dis.

[CR37] Shingler KL, Yoder JL, Carnegie MS, Ashley RE, Makhov AM, Conway JF, Hafenstein S (2013). The enterovirus 71 A-particle forms a gateway to allow genome release: a cryoEM study of picornavirus uncoating. PLoS Pathog.

[CR38] Singh S, Poh CL, Chow VT (2002). Complete sequence analyses of enterovirus 71 strains from fatal and non-fatal cases of the hand, foot and mouth disease outbreak in Singapore (2000). Microbiol Immunol.

[CR39] Spellman R, Rideau A, Matlin A, Gooding C, Robinson F, McGlincy N, Grellscheid SN, Southby J, Wollerton M, Smith CW (2005). Regulation of alternative splicing by PTB and associated factors. Biochem Soc Trans.

[CR40] Sweeney TR, Abaeva IS, Pestova TV, Hellen CU (2014). The mechanism of translation initiation on Type 1 picornavirus IRESs. EMBO J.

[CR41] Thompson SR, Sarnow P (2003). Enterovirus 71 contains a type I IRES element that functions when eukaryotic initiation factor eIF4G is cleaved. Virology.

[CR42] Tischendorf JJ, Beger C, Korf M, Manns MP, Kruger M (2004). Polypyrimidine tract-binding protein (PTB) inhibits Hepatitis C virus internal ribosome entry site (HCV IRES)—mediated translation, but does not affect HCV replication. Arch Virol.

[CR43] Tolbert M, Morgan CE, Pollum M, Crespo-Hernández CE, Li ML, Brewer G, Tolbert BS (2017). HnRNP A1 Alters the structure of a conserved enterovirus IRES domain to stimulate viral translation. J Mol Biol.

[CR44] Venkatramana M, Ray PS, Chadda A, Das S (2003). A 25 kDa cleavage product of polypyrimidine tract binding protein (PTB) present in mouse tissues prevents PTB binding to the 5′ untranslated region and inhibits translation of hepatitis A virus RNA. Virus Res.

[CR45] Wang J, Du J, Wu Z, Jin Q (2013). Quinacrine impairs enterovirus 71 RNA replication by preventing binding of polypyrimidine-tract binding protein with internal ribosome entry sites. PLoS ONE.

[CR46] Wei J, Ian EM, Gregory NF, Eileen S-CH, Gilbert JC (2000). Fibroblast growth factor receptor-1 alpha-exon exclusion and polypyrimidine tract-binding protein in glioblastoma multiforme tumors. Cancer Res.

[CR47] Witherell GW, Gil A, Wimmer E (1993). Interaction of polypyrimidine tract binding protein with the encephalomyocarditis virus mRNA internal ribosomal entry site. Biochemistry.

[CR48] Xie J, Lee JA, Kress TL, Mowry KL, Black DL (2003). Protein kinase A phosphorylation modulates transport of the polypyrimidine tract-binding protein. Proc Natl Acad Sci U S A.

[CR49] Zhu FC, Meng FY, Li JX, Li XL, Mao QY, Tao H, Zhang YT, Yao X, Chu K, Chen QH, Hu YM, Wu X, Liu P, Zhu LY, Gao F, Jin H, Chen YJ, Dong YY, Liang YC, Shi NM, Ge HM, Liu L, Chen SG, Ai X, Zhang ZY, Ji YG, Luo FJ, Chen XQ, Zhang Y, Zhu LW, Liang ZL, Shen XL (2013). Efficacy, safety, and immunology of an inactivated alum-adjuvant enterovirus 71 vaccine in children in China: a multicentre, randomised, double-blind, placebo-controlled, phase 3 trial. Lancet.

[CR50] Zhu F, Xu W, Xia J, Liang Z, Liu Y, Zhang X, Tan X, Wang L, Mao Q, Wu J, Hu Y, Ji T, Song L, Liang Q, Zhang B, Gao Q, Li J, Wang S, Hu Y, Gu S, Zhang J, Yao G, Gu J, Wang X, Zhou Y, Chen C, Zhang M, Cao M, Wang J, Wang H, Wang N (2014). Efficacy, safety, and immunogenicity of an enterovirus 71 vaccine in China. New Engl J Med.

