# Supplementary material for: Polypyrimidine Tract-Binding Protein Regulates Enterovirus 71 Translation Through Interaction with the Internal Ribosomal Entry Site
Source: Virol Sin. 2019 Feb 22;34(1):66–77. doi: 10.1007/s12250-019-00089-1 (PMC6420457; doi:10.1007/s12250-019-00089-1)
Supplement: Supplementary file 1 — Supplementary material 1 (PDF 80 kb) [file 12250_2019_89_MOESM1_ESM.pdf]

VIROLOGICA SINICA

Electronic Supplementary Material

Polypyrimidine Tract-Binding Protein Regulates Enterovirus 71 Translation through Interaction with the Internal Ribosomal Entry Site

Juemin Xi[1,#], Fei Ye[2,#], Guanzhou Wang[2], Wei Han[2], Zhizhong Wei[2], Bin Yin[2], Jiangang Yuan[2], Boqin Qiang[2], Xiaozhong Peng[1,2]✉

1. Institute of Medical Biology, Chinese Academy of Medical Sciences, and Peking Union Medical College, Kunming 650118, China
2. The State Key Laboratory of Medical Molecular Biology, Department of Molecular Biology and Biochemistry, Institute of Basic Medical Sciences, Chinese Academy of Medical Sciences and Peking Union Medical College, Beijing 100005, China

Supporting information to DOI: 10.1007/s12250-019-00089-1

Table S1. Primers used in the study.

Description	Primer	Sequence(5'–3')
pcDNA4-PTB	Fwd	TGGAATTCATGGACGGCA TTGTCCCAG
	Rev	CCGCTCGAGGATGGTGGACTTGGAG AAGGAGACC
pcDNA4-RRM12	Fwd	GGAATTCATGGACGGCA TTGTCCCAG
	Rev	CCGCTCGAGGAGCTTGGAA AAGTCGATG
pcDNA4-RRM34	Fwd	GGAATTCATGTC TGTATTGCTGGTCAGCAAC
	Rev	CCGCTCGAGGGACTTGGAG AAGGAGACC
pGEM-3zf-EV71 IRES	Fwd	CCGGAATTCCCCCCCAGTGAAACTTAGAAGCA GCAAACCACGATCA
	Rev	CCC AAGCTT GTTTAGCTGTGTTAAGGGTCAAG
pGEM-3zf-nt121-180	Fwd	GAATTCCCACGATCAATAGCGGGCAT
	Rev	AAGCTTGGGAAACAGAAGTGCTTGATCAAG
pGEM-3zf-nt190-230	Fwd	GAATTCTATCAATAGACTGCTCGCG
	Rev	AAGCTTCGAACGTTTTCTCCTTCAAC
pGEM-3zf-nt241-450	Fwd	GAATTCAGCTACTTCGGGAAACCTAG
	Rev	AAGCTTCGGAGGACTACTAACTAGC
pGEM-3zf-nt451-563	Fwd	GAATTCGCCCCTGAATGCGGCTAAT
	Rev	AAGCTTGAAACACGGACACCCAAAGTA
pGEM-3zf-nt564-742	Fwd	GAATTCTTTTTATCTTTATACTGGCTGCTTATG
	Rev	AAGCTTGTTTAGCTGTGTTAAGGGTC
pHRF-IRES	Fwd	ACGCGTCGACAAGCAGCAAACCACGATCAAT AGC
	Rev	TCCCCCGGGGTTTAGCTGTGTTAAGGGTCAAG
EV71-IRES	Fwd	AGCGGGTAGTGTGTCGTAAC G
	Rev	CGGATGGCCA ATCCAATAG
RPS16	Fwd	GCGCGGTGAGGTTGTCTAGTC
	Rev	GAGTTTTGAGTCACGATGGGC
